# AI Workflow, External Validation, and Development in Eye Disease Diagnosis

**DOI:** 10.1001/jamanetworkopen.2025.17204

**Published:** 2025-07-16

**Authors:** Qingyu Chen, Tiarnan D. L. Keenan, Elvira Agron, Alexis Allot, Emily Guan, Bryant Duong, Amr Elsawy, Benjamin Hou, Cancan Xue, Sanjeeb Bhandari, Geoffrey Broadhead, Chantal Cousineau-Krieger, Ellen Davis, William G. Gensheimer, Cyrus A. Golshani, David Grasic, Seema Gupta, Luis Haddock, Eleni Konstantinou, Tania Lamba, Michele Maiberger, Dimosthenis Mantopoulos, Mitul C. Mehta, Ayman G. Elnahry, Mutaz AL-Nawaflh, Arnold Oshinsky, Brittany E. Powell, Boonkit Purt, Soo Shin, Hillary Stiefel, Alisa T. Thavikulwat, Keith James Wroblewski, Yih Chung Tham, Chui Ming Gemmy Cheung, Ching-Yu Cheng, Emily Y. Chew, Michelle R. Hribar, Michael F. Chiang, Zhiyong Lu

**Affiliations:** 1 National Library of Medicine, National Institutes of Health, Bethesda, Maryland; 2 Department of Biomedical Informatics and Data Science, Yale School of Medicine, Yale University, New Haven, Connecticut; 3 National Eye Institute, National Institutes of Health, Bethesda, Maryland; 4 Yong Loo Lin School of Medicine, National University of Singapore, Singapore; 5 Save Sight Institute, Sydney University, Sydney, Australia; 6 Casey Eye Institute, Oregon Health & Science University, Portland; 7 VA White River Junction Healthcare System, White River Junction, Vermont; 8 Dartmouth Hitchcock Medical Center, Lebanon, New Hampshire; 9 Washington DC VA Medical Center, Washington, District of Columbia; 10 Carolina Vision Center, Fayetteville, North Carolina; 11 Bascom Palmer Eye Institute, University of Miami, Miami, Florida; 12 Krieger Eye Institute, Baltimore, Maryland; 13 Gavin Herbert Eye Institute, University of California, Irvine; 14 Fort Belvoir Community Hospital, Fort Belvoir, Virginia; 15 Uniformed Services University of the Health Sciences, Bethesda, Maryland; 16 George Washington University Hospital, The George Washington University, Washington, District of Columbia; 17 Department of Ophthalmology, Yong Loo Lin School of Medicine, National University of Singapore, Singapore; 18 Centre for Innovation and Precision Eye Health, Yong Loo Lin School of Medicine, National University of Singapore and National University Health System, Singapore; 19 Singapore Eye Research Institute, Singapore National Eye Centre, Singapore; 20 Ophthalmology and Visual Science Academic Clinical Program (Eye ACP), Duke-NUS Medical School, Singapore

## Abstract

**Importance:**

Timely disease diagnosis is challenging due to limited clinical availability and growing burdens. Although artificial intelligence (AI) has shown expert-level diagnostic accuracy, a lack of downstream accountability, including workflow integration, external validation, and further development, continues to hinder its clinical adoption.

**Objective:**

To address gaps in the downstream accountability of medical AI through a case study on age-related macular degeneration (AMD) diagnosis and severity classification.

**Design, Setting, and Participants:**

This diagnostic study developed and evaluated an AI-assisted diagnostic and classification workflow for AMD. Four rounds of diagnostic assessments (accuracy and time) were conducted with 24 clinicians from 12 institutions. Each round was randomized and alternated between manual (clinician diagnosis) and manual plus AI (clinician assisted by AI diagnosis), with a 1-month washout period. In total, 2880 AMD risk features were evaluated across 960 images from 240 Age-Related Eye Disease Study patient samples, both with and without AI assistance. For further development, the original DeepSeeNet model was enhanced into the DeepSeeNet+ model using 39 196 additional images from the US population and tested on 3 datasets, including an external set from Singapore.

**Exposure:**

Age-related macular degeneration risk features.

**Main Outcomes and Measures:**

The F1 score for accuracy (Wilcoxon rank sum test) and diagnostic time (linear mixed-effects model) were measured, comparing manual vs manual plus AI. For further development, the F1 score (Wilcoxon rank sum test) was again used.

**Results:**

Among 240 patients (mean [SD] age, 68.5 [5.0] years; 127 female [53%]), AI assistance significantly improved accuracy for 23 of 24 clinicians, increasing the mean F1 score from 37.71 (95% CI, 27.83-44.17) to 45.52 (95% CI, 39.01-51.61), with some improvements exceeding 50%. Manual diagnosis initially took an estimated 39.8 seconds (95% CI, 34.1-45.6 seconds) per patient, whereas manual plus AI saved 10.3 seconds (95% CI, −15.1 to −5.5 seconds) and remained faster by 6.9 seconds (95% CI, 0.2-13.7 seconds) to 8.6 seconds (95% CI, 1.8-15.3 seconds) in subsequent rounds. However, combining manual and AI did not always yield the highest accuracy or efficiency, underscoring challenges in explainability and trust. The DeepSeeNet+ model performed better in 3 test sets, achieving a significantly higher F1 score than the Singapore cohort (52.43 [95% CI, 44.38-61.00] vs 38.95 [95% CI, 30.50-47.45]).

**Conclusions and Relevance:**

In this diagnostic study, AI assistance was associated with improved accuracy and time efficiency for AMD diagnosis. Further development is essential for enhancing AI generalizability across diverse populations. These findings highlight the need for downstream accountability during early-stage clinical evaluations of medical AI.

## Key Points

**Question:**

How does artificial intelligence (AI) perform in the diagnosis of macular degeneration across clinicians, samples, and AI models?

**Findings:**

In this diagnostic study of 24 clinicians grading 2880 age-related macular degeneration risk features in 240 patients, AI assistance was associated with improved diagnostic accuracy by up to 50%, enhanced time efficiency, and broader generalization through further development. However, combining clinician diagnosis with AI assistance did not always yield the highest performance, especially compared with use of AI alone, underscoring challenges in explainability and clinician trust.

**Meaning:**

These findings suggest that systematic evaluations of AI workflows, external validation, and further development may be essential for closing accountability gaps and ensuring effective, generalizable medical AI.

## Teaser

This diagnostic study assesses downstream accountability of medical artificial intelligence (AI), focusing on AI-assisted workflow, external validation, and development, in age-related macular degeneration diagnosis and severity classification.

## Introduction

Timely disease diagnosis remains challenging due to rising disease burdens, limited clinician availability, and insufficient health care access.^1,2,3^ Although artificial intelligence (AI) has known potentials,^3,4,5,6^ many medical AI studies conclude after reporting testing set metrics alone, neglecting how AI integrates into clinical workflows, external validation for diverse populations, and continual algorithmic refinement.^7,8,9,10,11^

The aim of our study was to address these gaps through a case study of age-related macular degeneration (AMD), a leading cause of vision loss.^12,13,14,15,16^ We developed an AI-assisted diagnostic workflow and conducted 4 rounds of assessments to measure both accuracy and efficiency, comparing performance with and without AI assistance. We also refined the AI algorithm using additional medical images and tested it on 3 benchmarks, including an external cohort. Overall, our study underscores the importance of downstream accountability during early-stage clinical evaluations of medical AI, moving beyond reliance on internal benchmarking test sets alone. The code and models are publicly available.^17^

## Methods

### Clinical Standard of AMD Diagnosis

This diagnostic study received National Institutes of Health Institutional Review Board approval, and written informed consent for the research was obtained from all study participants. The study followed the Standards for Reporting of Diagnostic Accuracy Studies (STARD) reporting guideline.

The current standard of care for diagnosing AMD in clinical practice and performing prognostic stratification for risk of progression to late AMD is the Age-Related Eye Diseases Study (AREDS) Simplified Severity Scale.^18^ The diagnostic and severity classification procedure comprises 2 steps. First, 3 macular risk features are identified and categorized (individual risk factors) separately for both eyes: drusen size (ranging from 0 to 2), pigmentary abnormalities (ranging from 0 to 1), and late AMD (ranging from 0 to 1). Second, these individual risk feature scores for both eyes are used to calculate the overall AMD severity level for that individual on a scale of 0 to 5. The quantification and interpretation of each severity level is summarized in eTable 1 in Supplement 1.

### Clinician Participants and the AI Model

The AI-assisted diagnostic workflow was designed to support clinicians in diagnosing and classifying the severity of AMD. This study conducted multiround and head-to-head comparisons involving AI and 24 clinicians from 12 institutions to evaluate both diagnostic accuracy and time efficiency with and without AI assistance.

DeepSeeNet^5^ was selected as the AI model to assist in the diagnosis and severity classification of AMD within the workflow because of its state-of-the-art performance and free availability. This model was trained and validated on approximately 60 000 images from a longitudinal study of approximately 4500 individuals, representing a broad range of AMD severity from none to advanced disease.

The clinicians were recruited to participate in the evaluation of AI-assisted diagnostic and severity classification workflow. The clinicians included 13 retina specialists and 11 ophthalmologists who were not retina specialists (ie, general ophthalmology and other subspecialties) (eTable 2 in Supplement 1).

### Design and Implementation of the AI-Assisted Diagnostic Workflow

**Figure 1.**
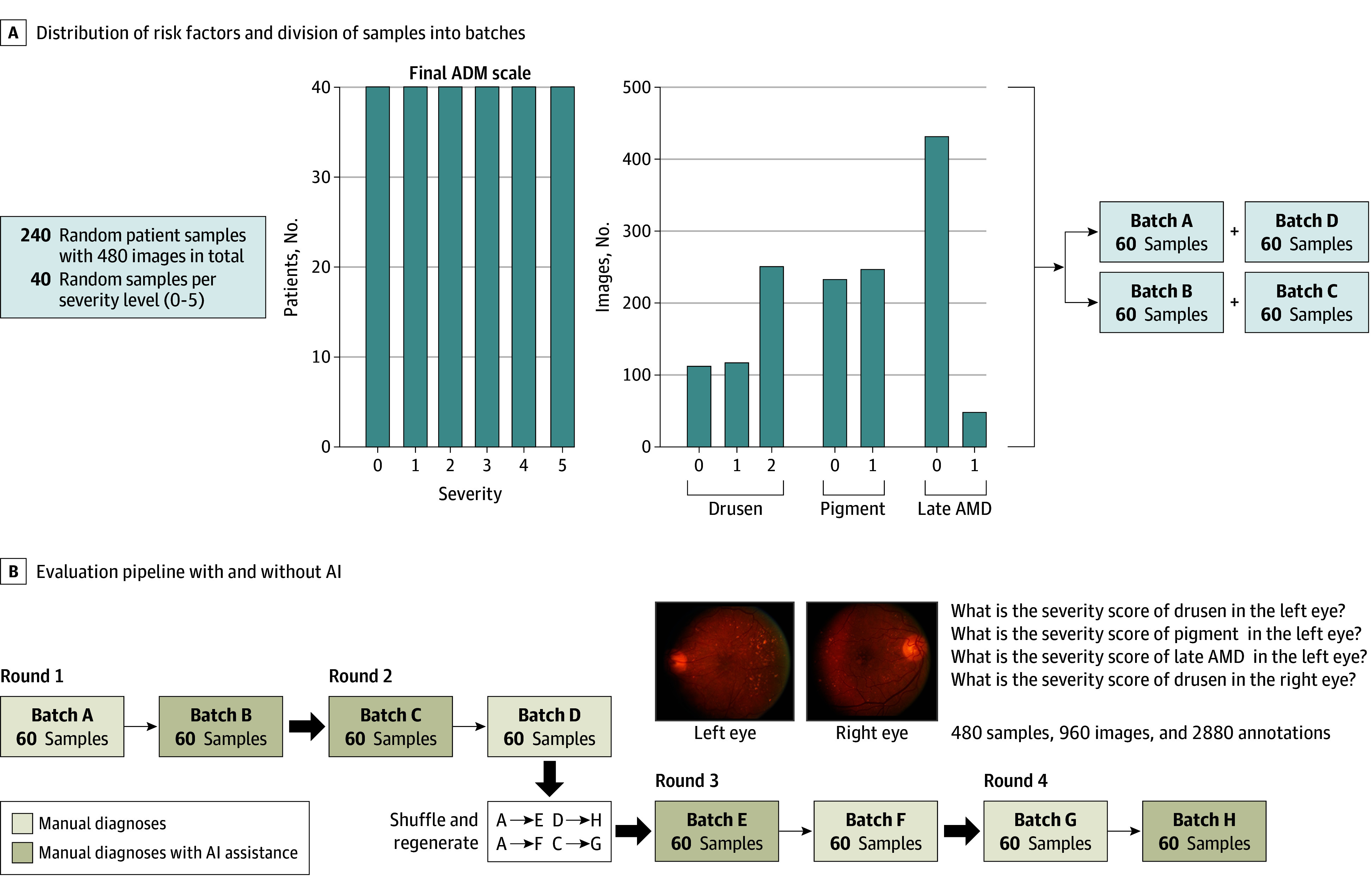
Overview of the Artificial Intelligence (AI)–Assisted Diagnostic/Classification Workflow AMD indicates age-related macular degeneration.

Figure 1 illustrates the AI-assisted diagnostic workflow. To assess the effectiveness of the AI-assisted AMD diagnosis and severity classification workflow, we conducted a comprehensive comparative analysis. We first created an evaluation dataset from the test set of the DeepSeeNet model (Figure 1A). The criterion standard labels originated from expert human grading of the images at the Wisconsin Reading Center, as described previously in detail.^5,19^

In brief, 2 human experts graded the images for the AMD risk features, then a computerized algorithm calculated the severity levels. In the case of any discrepancy regarding the severity level between graders, a senior investigator would adjudicate the final severity level. To create the evaluation dataset, we randomly selected 40 samples for each AMD severity level, ranging from 0 to 5, ie, to generate an equally distributed dataset. The resulting dataset included 240 patient samples comprising 480 color fundus photographs. Subsequently, we randomly divided these samples into 4 separate batches and ensured that images from each patient were present in only 1 batch (Figure 1B).

The comparative analysis was completed in 4 rounds (Figure 1C). Each round comprised 2 batches: 1 in which clinicians annotated images manually (eg, batch A), referred to as manual, and 1 in which clinicians annotated with AI assistance (eg, batch B), referred to as manual plus AI. Clinicians provided their final diagnosis and severity scores in both scenarios, while their diagnostic times were tracked. The order of manual vs manual plus AI was reversed in each pair of rounds (eg, rounds 1 vs 2 and rounds 3 vs 4).

After the first 2 rounds, each clinician graded all samples exactly once, with half under manual and the other half under manual plus AI. At this point, a washout period of 1 month was introduced, the batches were renamed (batch A was renamed batch E, etc), the samples within each batch were randomly reordered, and their identifiers were changed to prevent memorization and bias. The same clinicians then repeated the process in another 2 rounds, with batches that had been presented as manual now presented as manual plus AI and vice versa (eg, batch A, initially manual, became batch E under manual plus AI) (Figure 1C). Additionally, the order of presentation was reversed (eg, if round 1 started with manual, round 3 started with manual plus AI) (Figure 1C). By the end, each clinician graded all samples exactly twice, once under manual and once under manual plus AI.

### Evaluation Measures

The evaluation assessed whether AI assistance could enhance both the effectiveness and time efficiency of diagnostic and severity classification. Effectiveness was quantified using the F1 score as the primary metric, complemented by precision, specificity, and sensitivity. Efficiency was measured by the time taken in seconds per patient for diagnosis.

### Further Development and External Validations

We further curated an additional 39 916 fundus images from 2940 participants with AMD in the AREDS2 and split these into 70%, 10%, and 20% for training, validation, and testing sets, respectively. The AREDS2 was a multicenter, phase 3 randomized clinical trial designed to enroll individuals at moderate to high risk of progression to late AMD.^20,21^ We combined the 2 training sets (ie, from AREDS and AREDS2) to perform additional training on the model, then compared the performance of the new model (named DeepSeeNet+) with the original model (DeepSeeNet) on 3 test sets: (1) the 240 patient samples from the original AREDS testing set (referred to as the AREDS set); (2) 150 patient samples with a simplified AREDS scale level of 3 to 5 (50 patients per level), focused on intermediate and late AMD, from the testing set of the AREDS2 dataset (referred to as the AREDS2 set); and (3) 180 patient samples with a simplified AREDS scale level of 1 to 5 from the Singapore Epidemiology of Eye Diseases (SEED) Study^22^ (referred to as the SEED set).

The SEED Study is a multiethnic, population-based study aimed at providing insights into the epidemiology of eye diseases across 3 major ethnic groups (Malay, Indian, and Chinese) in Singapore. We used this set to evaluate the performance of the models on external populations. The training details are provided in the eMethods in Supplement 1.

### Statistical Analysis

#### Quantifying AI-Assisted Effectiveness

A 2-sided Wilcoxon rank sum test (*P* < .05 considered significant) was used to compare the F1 scores for manual and manual plus AI. All analyses were conducted using the SciPy, version 1.15.2 package in Python, version 3.13.2 (Python Software Foundation).

#### Quantifying AI-Assisted Time Efficiency

Each clinician participated in 4 rounds, annotating images both manually and with AI assistance while their diagnostic times were recorded. To minimize confounding factors, we alternated the order of manual vs AI-assisted tasks in each round. Nevertheless, within-clinician correlation and practice effects may still occur. To address these, we used a linear mixed-effects model (*P* < .05 considered significant) with random intercepts for each clinician, treating round (1-4), method (manual vs manual plus AI), and their interaction as fixed effects. The analysis was conducted using the statsmodels, version 0.14.4 package in Python, version 3.13.2.

#### Quantifying External Validation Performance

For each of 3 test sets, we performed bootstrapping with a random sample size of 60 (drawn without replacement) over 100 iterations for both DeepSeeNet and DeepSeeNet+. We then applied a 2-sided Wilcoxon rank sum test (*P* < .05 considered significant) to compare their F1 scores. The analysis was performed using the SciPy, version 1.15.2 package in Python, version 3.13.2.

## Results

From the AREDS study, we selected 240 patients by randomly choosing 40 samples for each AMD severity level (0-5). The patients’ mean (SD) age was 68.5 (5.0) years, and 127 were female (53%) and 113 male (47%).

The 4-round AI-assisted diagnostic workflow was conducted with 24 clinician participants from 12 institutions. All 24 clinicians’ annotations were properly captured in each round. However, diagnostic times were only recorded across all 4 rounds for 19 clinicians, as 5 clinicians had missing data in 1 of the rounds.

### Incorporating AI Into the Clinical Workflow

**Figure 2.**
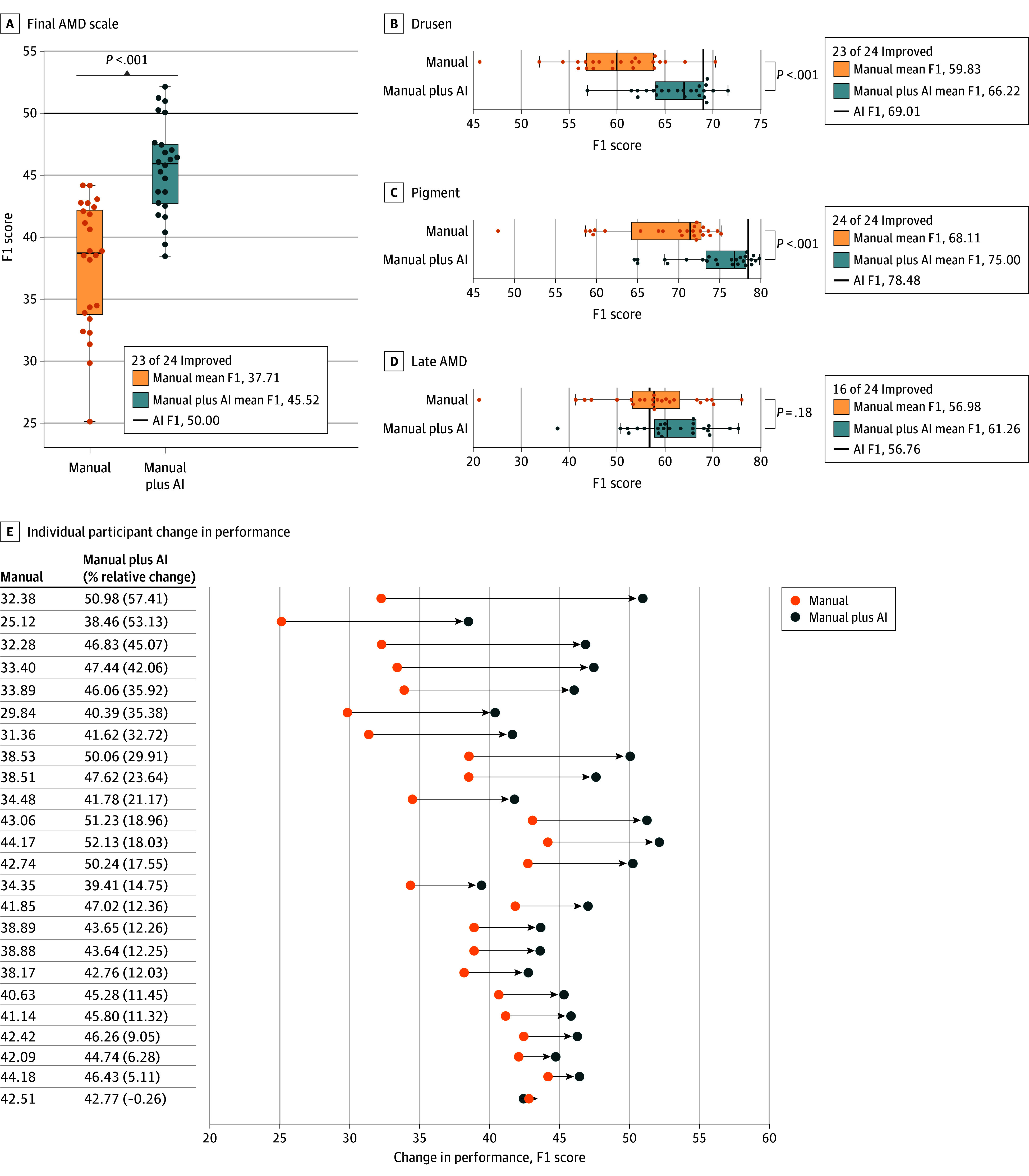
Comparison of Diagnostic Performance (F1 Score) for Manual vs Manual Plus Artificial Intelligence (AI) Assessment Each dot represents an F1 score. Solid black cutoff lines represent the performance of the AI model alone. A-D, The bar inside the boxes indicates the median, and the lower and upper ends of the boxes are the first and third quartiles. The whiskers indicate values within 1.5-times the IQR from the upper or lower quartile (or the minimum and maximum if within 1.5-times the IQR of the quartiles). AMD indicates age-related macular degeneration.

Figure 2 details the diagnostic and classification performance, comparing F1 scores for both manual and manual plus AI scenarios. Figure 2A illustrates the overall AMD severity level. Overall, the results indicated an improvement in manual AMD diagnostic and classification accuracy with the AI assistance in 23 of the 24 clinicians. Specifically, the mean F1 score for grading AMD severity increased from 37.71 (95% CI, 27.83-44.17) to 45.52 (95% CI, 39.01-51.61) (*P* < .001). Secondary evaluation metrics also showed that manual plus AI consistently outperformed manual (eTable 3 in Supplement 1).

For drusen size grading (Figure 2B), the mean F1 score increased with AI assistance from 59.83 (95% CI, 49.25-68.45) to 66.22 (95% CI, 59.54-70.69) (*P* < .001), with 23 of 24 clinicians demonstrating higher diagnostic accuracy when using AI assistance. Similarly, for pigmentary abnormality grading (Figure 2C), the mean F1 score increased from 68.11 (95% CI, 54.14-74.91) to 75.00 (95% CI, 64.83-79.56) (*P* < .001), with 23 of 24 clinicians showing higher diagnostic accuracy with AI assistance. Finally, for late AMD grading (Figure 2D), the mean F1 score increased from 56.98 (95% CI, 32.82-72.62) to 61.26 (95% CI, 45.11- 74.30) (*P* = .18), with 16 of 24 clinicians showing higher diagnostic accuracy with AI assistance. Performance of AI alone produced higher F1 scores than manual plus AI for 19 of 24 clinicians (drusen size grading, 17 of 24 clinicians; pigmentary abnormality grading, 19 of 24 clinicians). However, 18 of 24 clinicians achieved higher performance than AI alone for late AMD grading.

Figure 2E shows the diagnostic performance changes of the overall AMD severity scale at the individual clinician grader level. It shows that the F1 score of individual clinicians on the overall AMD severity scale improved with AI assistance over a range of 5.11% to 57.41%, with 23 showing higher diagnostic accuracy when using AI assistance. There was only 1 instance where the performance of manual plus AI did not improve, but the difference was only 0.26% lower, with the F1 score changing from 42.77 to 42.51.

**Figure 3.**
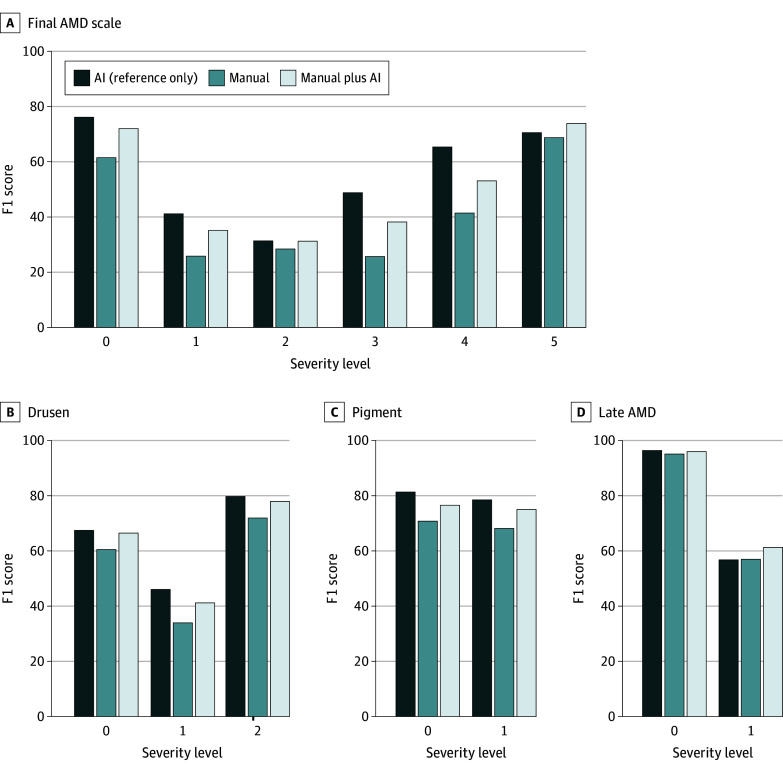
Detailed Breakdown of F1 Score per Scale Manual and manual plus artificial intelligence (AI) results represent paired comparisons from the same clinicians. The AI-only performance is from a single model and is shown for reference only; it is not directly comparable with the clinician results. AMD indicates age-related macular degeneration.

Figure 3 provides more detailed performance results for manual vs manual plus AI at each severity level in the overall AMD severity level (Figure 3A) and its 3 constituent risk features (Figure 3B-D). The results show that manual accuracy was improved with AI assistance consistently at each severity level. Notably, for the overall AMD severity level, the largest improvement was observed in the final AMD severity scale of 3, in which the F1 score was enhanced with AI assistance from 23.09 to 34.36 and may be largely due to the improved accuracy of drusen size and pigmentary abnormality grading with AI assistance. The F1 score of medium drusen and large drusen improved from 33.94 to 41.17 and from 71.90 to 77.89, respectively. Similarly, the F1 score of the presence of pigment improved from 68.11 to 75.00. In contrast, for grading late AMD presence, while its F1 score improved from 56.98 to 61.26 with the AI assistance, performance was still relatively lower than that of the other 2 risk factors.

**Figure 4.**
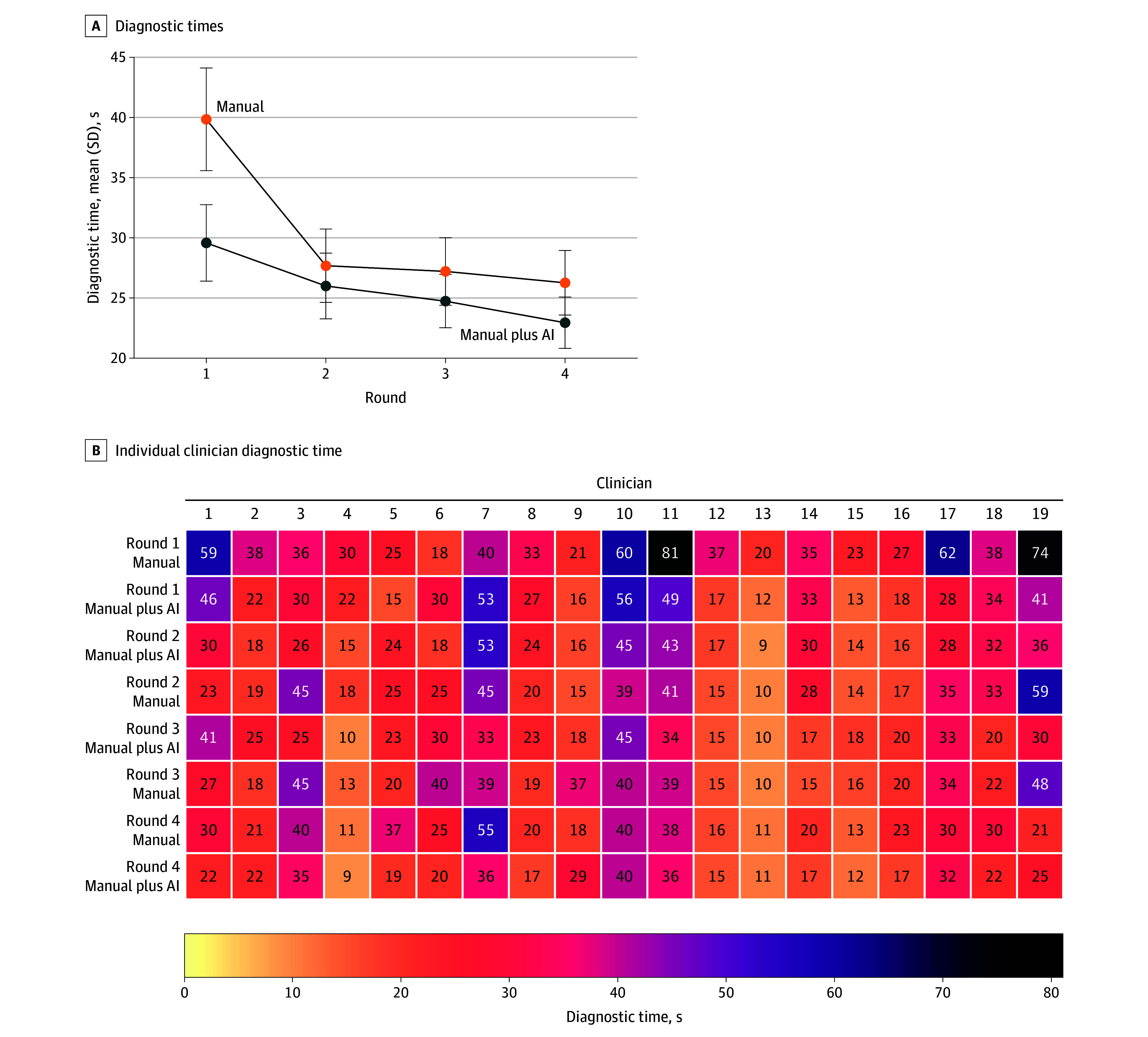
Diagnostic Time Efficiency With Artificial Intelligence (AI) Assistance

The diagnostic time was recorded properly in all 4 rounds for 19 of the 24 clinician participants. Figure 4A illustrates the mean diagnostic times for manual and manual plus AI across these rounds, while Figure 4B shows a more granular, physician-level breakdown. Detailed results are available in eTable 4 in Supplement 1. In round 1, manual required an estimated 39.8 seconds (95% CI, 34.1-45.6 seconds); AI assistance reduced this by 10.3 seconds (95% CI, −15.1 to −5.5 seconds) (*P* < .001). Although manual times improved by approximately 12 to 14 seconds in rounds 2 to 4 (indicating a learning curve or practice effect), AI assistance remained significantly faster by 6.9 seconds (95% CI, 0.2-13.7 seconds; *P* = .04) to 8.6 seconds (95% CI, 1.8-15.3 seconds; *P* = .01) in each subsequent round. Overall, these findings suggest that AI assistance provides a meaningful reduction in diagnostic time, even after accounting for practice effects over repeated testing. Note that other factors may also influence these results. For instance, the random intercept variance was 108.3, indicating substantial baseline differences in clinicians’ speeds.

### Further Development

The Table shows performance across the 3 external testing sets of both models. The results reveal that DeepSeeNet+ had superior performance over the AREDS2 set and the SEED set, with an F1 score of 64.95 (95% CI, 48.73-79.47) vs 51.62 (95% CI, 37.38-64.47) (*P* < .001) and 52.43 (95% CI, 44.38-61.00) vs 38.95 (95% CI, 30.50-47.45) (*P* < .001), respectively, and had superior performance at almost all severity levels. For the AREDS set that was used in the evaluation of AI-assisted diagnostic workflow (the same distribution of the training set from the original AI model), the F1 score remained the same.

**Table.** F1 Scores for Diagnosing AMD Using the DeepSeeNet and DeepSeeNet+ Models Across 3 Datasets: AREDS, AREDS2, and SEED

Final AMD scale	F1 score, mean (95% CI)^a^		*P* value
	DeepSeeNet model	DeepSeeNet+ model	
**AREDS**			
Overall	0.4755 (0.3066-0.6434)	0.4793 (0.3091-0.6554)	.95
0	0.6852	0.6667	
1	0.3704	0.3797	
2	0.2821	0.2927	
3	0.4390	0.3421	
4	0.5882	0.5833	
5	0.6349	0.7302	
**AREDS2**			
Overall	0.5162 (0.3738-0.6447)	0.6395 (0.4873-0.7947)	<.001
3	0.4211	0.4872	
4	0.4091	0.6491	
5	0.7391	0.8163	
**SEED**			
Overall	0.3895 (0.3050-0.4745)	0.5243 (0.4438-0.6100)	<.001
0	0.5915	0.6275	
1	0.3125	0.5000	
2	0.2609	0.1923	
3	0.3396	0.4478	
4	0.3158	0.7077	
5	0.5538	0.7385	

Abbreviations: AMD, age-related macular degeneration; AREDS, Age-Related Eye Diseases Study; SEED, Singapore Epidemiology of Eye Diseases.

^a^ Scores are detailed for each severity scale (0-5), comparing performance between the 2 models.

## Discussions

This diagnostic study had 3 primary findings. First, AI assistance was associated with both improved manual diagnostic and classification accuracy and time efficiency. Specifically, we found that AI assistance was associated with enhanced grading performance for overall AMD severity and individual AMD risk features among both retina specialists and comprehensive ophthalmologists. In terms of efficiency, AI assistance was significantly associated with reduced diagnostic times in the first round, with the benefit decreasing but remaining evident in later rounds after accounting for practice effects over repeated testing. Collectively, these findings show the potential of integrating AI assistance into clinical practice.

Second, while manual plus AI often outperformed manual alone, it did not necessarily exceed the performance of AI alone. As shown in Figure 2, for overall AMD severity, AI alone produced higher F1 scores than manual plus AI in 19 of 24 cases. For drusen size grading, AI alone had higher F1 scores in 17 of 24 cases, and for pigmentary abnormality grading, AI alone exceeded manual plus AI in 19 of 24 cases. In contrast, for late AMD grading, 18 of 24 clinicians achieved higher performance than AI alone. This finding highlights the ongoing challenges in making medical AI algorithms explainable and trustworthy for clinicians.

Third, a systematic comparison between DeepSeeNet and DeepSeeNet+ revealed that the latter had superior performance, particularly on external populations. This finding underscores the importance of continued development and refinement of existing medical AI algorithms.

Previous studies have highlighted the importance of downstream accountability in AI-assisted eye disease diagnosis, citing limited external validations, minimal evaluations with ophthalmologists, and insufficient ongoing monitoring.^7,8,9,10,11^ For instance, 1 study reported that AI-assisted diabetic retinopathy diagnosis had a positive predictive value of only 12% in 193 patients during external validation.^23^ Although multiple studies have compared AI performance directly with that of ophthalmologists,^5,6,24,25^ very few have assessed whether AI assistance actually improves clinicians’ diagnostic accuracy or time efficiency. In particular, the Collaborative Community on Ophthalmic Imaging Working Group pointed out that benchmarking the performance of a combined human and AI model workflow is understudied.^26^

Beyond ophthalmology, downstream accountability of AI in health care has consistently been a concern.^27,28,29,30^ A comprehensive review of 82 studies on AI disease classification algorithms across 18 specialties (eg, cardiology, respiratory, ophthalmology) stressed that only 14 studies compared performance with that of health care professionals and only 25 included external validation.^7^ Similar observations have also been reported in other reviews.^8,9,10,11^ Importantly, of the 14 studies, none evaluated how AI might assist health care professionals in improving diagnostic accuracy and time efficiency, an essential goal since AI disease classification algorithms are primarily intended to support clinical expertise.

Studies have further shown that there is little evidence for improved clinician performance with AI assistance.^31,32^ Such issues have been highlighted by the Developmental and Exploratory Clinical Investigations of Decision Support Systems Driven by Artificial Intelligence (DECIDE-AI), which provides expert consensus statements on the reporting guidelines for early-stage clinical evaluation.^33^ Our study may help fill this gap in downstream accountability by showing precisely how AI assistance could enhance manual diagnosis and be integrated into clinical workflows. Notably, while AI assistance holds promise for improving diagnostic workflows, challenges, such as ensuring the highest possible accuracy and efficiency through manual plus AI, remain. These findings illustrate why early-stage clinical evaluations of medical AI must extend beyond internal benchmarking test sets and include thorough clinical assessments of how AI assists clinicians.

### Limitations

Our study has several limitations. First, the evaluation of the AI-assisted disease diagnosis workflow used samples from the test set of the original AI model, which may have led to an overestimation of AI performance. Nevertheless, as shown in the Table, its overall performance on 3 independent datasets, including the test set, remained similar. In the future, we aim to use data from new patients with consent for further evaluations.

Second, although we have implemented a stringent evaluation study design and minimized potential biases, AMD may still be challenging to grade based on color fundus photographs alone, which might result in lower overall diagnostic accuracy. We will explore additional imaging modalities and tools to enhance performance in clinical scenarios.

## Conclusions

In this diagnostic study involving 24 clinicians from 12 institutions diagnosing 2880 AMD risk features in 240 patients across multiple rounds (with and without AI assistance), the results show that AI assistance was associated with both improved accuracy and time efficiency. Following additional training, the AI model also showed broader generalizability across diverse populations. Nonetheless, challenges such as algorithm explainability and clinician trust remain. The results illustrate the need for downstream accountability during early-stage clinical evaluations of medical AI beyond relying solely on internal benchmarking and highlight the importance of ongoing validation and refinement to ensure broader applicability in clinical practice and ultimately facilitate more effective disease management.

## Notes

Supplement 1. **eTable 1.** Clinical classification of Age-Related Macular Degeneration **eTable 2.** Participant Details **eMethods.** Training Details **eTable 3.** Detailed Evaluation Results Between Manual and Manual Plus AI **eTable 4.** Detailed Mixed Linear Model Regression Results

Supplement 2.
Data Sharing Statement
