# Supplementary material for: AI Workflow, External Validation, and Development in Eye Disease Diagnosis
Source: JAMA Netw Open. 2025 Jul 16;8(7):e2517204. doi: 10.1001/jamanetworkopen.2025.17204 (PMC12268484; doi:10.1001/jamanetworkopen.2025.17204)
Supplement: Supplement 1. — eTable 1. Clinical classification of Age-Related Macular Degeneration eTable 2. Participant Details eMethods. Training Details eTable 3. Detailed Evaluation Results Between Manual and Manual Plus AI eTable 4. Detailed Mixed Linear Model Regression Results [file jamanetwopen-e2517204-s001.pdf]

# Supplemental Online Content

Chen Q, Keenan TDL, Agron E, et al. Accountable AI in eye disease diagnosis workflow, external validation, and development. *JAMA Netw Open*. 2025;8(6):e2517204. doi:10.1001/jamanetworkopent.2025.17204

**eTable 1.** Clinical classification of Age-Related Macular Degeneration

**eTable 2.** Participant Details

**eMethods.** Training Details

**eTable 3.** Detailed Evaluation Results Between Manual and Manual Plus AI

**eTable 4.** Detailed Mixed Linear Model Regression Results

This supplemental material has been provided by the authors to give readers additional information about their work.

© 2025 Chen Q et al. *JAMA Network Open*.

**eTable 1.** Clinical Classification of Age-Related Macular Degeneration

| | Scale | Interpretation | Quantification | Treatment/Follow-up procedure |
|---|---|---|---|---|
| **1. Individual risk factors** | | | | |
| Macular drusen | 0 | No or small drusen | Drusen size within 63 µm | No AMD present |
| | 1 | Medium drusen | Drusen size between 63 µm and 125 µm | Early AMD; home monitoring of vision by Amsler grid; repeat examination/imaging in 6-24 months |
| | 2 | Large drusen | Drusen size over 125 µm | Intermediate AMD; AREDS2 oral supplements; home monitoring of vision by Amsler grid or electronic device; repeat examination/imaging in 6-18 months |
| Pigmentary abnormalities | 0 | Absent | | Depends on drusen status |
| | 1 | Presence of hyper/hypopigmentary abnormalities | | Intermediate AMD; AREDS2 oral supplements; home monitoring of vision by Amsler grid or electronic device; repeat examination/imaging in 6-18 months |
| Late AMD | 0 | None | | Depends on drusen/pigmentary abnormality status |
| | 1 | Presence of neovascular AMD and/or any geographic atrophy | | Prompt treatment with anti-VEGF therapy for neovascular AMD; observation or possible treatment with complement inhibitor therapy for geographic atrophy |
| **2. Final AMD scale** | 0 | | No large drusen or pigmentary abnormalities in either eye | Early AMD or no AMD; see above for recommendations |
| | 1 | | Either large drusen or pigmentary abnormalities in one eye only | Intermediate AMD with 3% risk of progression to late AMD in either eye at 5 years; see above for recommendations |
| | 2 | | Large drusen and/or pigmentary abnormalities between both eyes (either both features in one eye or one feature in each eye) | Intermediate AMD with 12% risk of progression to late AMD in either eye at 5 years; see above for recommendations |
| | 3 | | Three risk features between both eyes | Intermediate AMD with 25% risk of progression to late AMD in either eye at 5 years; see above for recommendations |
| | 4 | | Four risk features between both eyes | Intermediate AMD with 50% risk of progression to late AMD in either eye at 5 |

© 2025 Chen Q et al. *JAMA Network Open*.

| | | | |
|---|---|---|---|
| | | | years; see above for recommendations |
| 5 | Late AMD | Neovascular AMD and/or any geographic atrophy in at least one eye | Prompt treatment with anti-VEGF therapy for neovascular AMD; observation or possible treatment with complement inhibitor therapy for geographic atrophy |

© 2025 Chen Q et al. *JAMA Network Open*.

**eTable 2.** Participant Details

| Name | Expertise | Affiliation | Email |
|---|---|---|---|
| Cyrus Golshani | retina | VA | cyrus.golshani@va.gov |
| Brittany E. Powell | retina | Navy/Fort Belvoir Community Hospital | brittany.e.powell.mil@health.mil |
| David Grasic | retina | Carolina Vision Center | david_grasic@hotmail.com |
| Alisa Thavikulwat | retina | NIH | alisa.thavikulwat@nih.gov |
| Mitul Mehta | retina | UCI | mcmehta@hs.uci.edu |
| Dimosthenis Mantopoulos | retina | Dartmouth, Geisel School of Medicine | dmanto@hitchcock.org |
| Tiarnan Keenan | retina | NIH | tiarnan.keenan@nih.gov |
| Sanjeeb Bhandari | fellow | NIH/Vision Innovation Partners | sbha5189@uni.sydney.edu.au |
| Geoffrey Broadhead | fellow | Sydney University | geoffrey.broadhead@sydney.edu.au |
| Luis Haddock | retina | University of Miami- Bascom Palmer Eye Institute | ljhaddock@med.miami.edu |
| Soo Shin | non-retina | VA | soo.shin@va.gov |
| Boonkit Purt | non-retina | Army/USUHS | boonkit.purt.mil@health.mil |
| Michele Maiberger | non-retina | VA | michele.maiberger@va.gov |
| William G Gensheimer | non-retina | 1. White River Junction VA Medical Center 2. Geisel School of Medicine at Dartmouth | William.Gensheimer@va.gov |
| Arnold Oshinsky | non-retina | VA | Arnold.Oshinsky@va.gov |
| Tania Lamba | non-retina | Krieger Eye Institute | tania_lamba@yahoo.com |
| Chantal Cousineau-Krieger | non-retina | NIH | chantal.cousineaukrieger@nih.gov |
| Keith James Wroblewski | non-retina | GWU | kwroblewski@mfa.gwu.edu |
| Ellen Davis | non-retina | OHSU | davise@ohsu.edu |
| Hillary Stiefel | non-retina | OHSU | stiefel@ohsu.edu |
| Seema Gupta | non-retina | OHSU | guptase@ohsu.edu |
| Ayman nahri | fellow | NIH | ayman_elnahri@hotmail.com |
| Mutaz Nawafleh | fellow | NIH | mutazoph@gmail.com |
| Eleni Konstantinou | fellow | NIH | eleni.konstantinou@gmail.com |

© 2025 Chen Q et al. *JAMA Network Open*.

**eMethods.** Training Details

The DeepSeeNet+ model was trained on a machine equipped with NVIDIA Tesla V100 GPUs, TensorFlow (v2.9.1) were used for implementation in Python (v3.8). To make a direct comparison, we followed the original DeepSeeNet implementation where possible. The Inception23 was used as the backbone model. The Adam optimizer was set with a learning rate of [0.0001]. Training was conducted over [50 epochs with a batch size of 16] where early stop of 5 was used to prevent overfitting. Data augmentation techniques, including rotations and flips, were employed to enhance model performance.

© 2025 Chen Q et al. *JAMA Network Open*.

**eTable 3.** Detailed Evaluation Results Between Manual and Manual Plus AI

Comparison of Diagnostic Accuracy Measures Between Manual Assessment and AI-Assisted Manual Assessment for Age-Related Macular Degeneration (AMD) Diagnosis: (A) AMD Severity Scale and (B) Individual Risk Features. Results include F1 score, precision, specificity, and sensitivity, along with statistical significance (P-value). The percentage of relative change is indicated in brackets. Confidence intervals for the primary evaluation metric F1-score are also provided.

**(A) Overall accuracy**

| | | Manual | Manual + AI | | P-value |
|---|---|---|---|---|---|
| **Final AMD scale** | **23 out of 24 improved** | | | | |
| | F1 | 0.3771 (0.2783-0.4417) | 0.4552 (0.3901-0.5161) | (+20.69%) | < .001 |
| | Precision | 0.3944 | 0.4717 | (+19.60%) | |
| | Specificity | 0.8784 | 0.8932 | (+1.68%) | |
| | Sensitivity | 0.3922 | 0.4660 | (+18.81%) | |

**(B) Risk factor accuracy**

| | | Manual | Manual + AI | | P-value |
|---|---|---|---|---|---|
| **Drusen** | **23 out of 24 improved** | | | | |
| | F1 | 0.5998 (0.4925-0.6845) | 0.6627 (0.5954-0.7069) | (+10.49%) | < .001 |
| | Precision | 0.6440 | 0.6972 | (+8.25%) | |
| | Specificity | 0.8126 | 0.8518 | (+4.83%) | |
| | Sensitivity | 0.5971 | 0.6549 | (+9.67%) | |
| **Pigment** | **24 out of 24 improved** | | | | |
| | F1 | 0.6811 (0.5414-0.7491) | 0.7500 (0.6483-0.7956) | (+10.11%) | < .001 |
| | Precision | 0.7546 | 0.8082 | (+7.11%) | |
| | Specificity | 0.7618 | 0.8131 | (+6.74%) | |
| | Sensitivity | 0.6417 | 0.7088 | (+10.46%) | |
| **Late AMD** | **16 out of 24 improved** | | | | |
| | F1 | 0.5698 (0.3282-0.7262) | 0.6126 (0.4511-0.7430) | (+7.52%) | 0.18 |
| | Precision | 0.6241 | 0.6999 | (+12.16%) | |
| | Specificity | 0.9535 | 0.9676 | (+1.48%) | |
| | Sensitivity | 0.3922 | 0.4660 | (+18.81%) | |

© 2025 Chen Q et al. *JAMA Network Open*.

**eTable 4.** Detailed Mixed Linear Model Regression Results

This section presents the full regression outputs for the linear mixed-effects model, where diagnostic time (seconds per patient) serves as the outcome variable. The model includes Method (Manual vs. Manual + AI) and Round (1–4) as fixed effects, and random intercepts for each clinician.

Mixed Linear Model Regression Results

| | | | |
|---|---|---|---|
| Model: | MixedLM | Dependent Variable: | Result |
| No. Observations: | 152 | Method: | REML |
| No. Groups: | 19 | Scale: | 56.6571 |
| Min. group size: | 8 | Log-Likelihood: | -531.8876 |
| Max. group size: | 8 | Converged: | Yes |
| Mean group size: | 8.0 | | |

| | Coef. | Std.Err. | z | P>\|z\| | [0.025 | 0.975] |
|---|---|---|---|---|---|---|
| Intercept | 39.842 | 2.947 | 13.521 | 0.000 | 34.067 | 45.617 |
| Method[T.Manual + AI] | -10.263 | 2.442 | -4.203 | 0.000 | -15.050 | -5.477 |
| Factor[T.Round 2] | -12.158 | 2.442 | -4.978 | 0.000 | -16.944 | -7.371 |
| Factor[T.Round 3] | -12.632 | 2.442 | -5.172 | 0.000 | -17.418 | -7.845 |
| Factor[T.Round 4] | -13.579 | 2.442 | -5.560 | 0.000 | -18.365 | -8.792 |
| Method[T.Manual + AI]:Factor[T.Round 2] | 8.579 | 3.454 | 2.484 | 0.013 | 1.810 | 15.348 |
| Method[T.Manual + AI]:Factor[T.Round 3] | 7.789 | 3.454 | 2.255 | 0.024 | 1.020 | 14.559 |
| Method[T.Manual + AI]:Factor[T.Round 4] | 6.947 | 3.454 | 2.012 | 0.044 | 0.178 | 13.716 |
| Group Var | 108.318 | 5.463 | | | | |

© 2025 Chen Q et al. *JAMA Network Open*.
