# Supplementary material for: AI Workflow, External Validation, and Development in Eye Disease Diagnosis
Source: JAMA Netw Open. 2025 Jul 16;8(7):e2517204. doi: 10.1001/jamanetworkopen.2025.17204 (PMC12268484; doi:10.1001/jamanetworkopen.2025.17204)
Supplement: Supplement 2. — Data Sharing Statement [file jamanetwopen-e2517204-s002.pdf]

Data Sharing Statement

Chen. Accountability of AI in Eye Disease Diagnosis Workflow, External Validation, and Development. *JAMA Netw Open*. Published July 09, 2025.
doi:10.1001/jamanetworkopen.2025.17204

Data
Data available: Yes
Data types: Participant data with identifiers
How to access data: The AREDS data set (NCT00000145) generated during and/or analyzed during the current study is available in the dbGAP repository, https://www.ncbi.nlm.nih.gov/projects/gap/cgi-bin/study.cgi?study_id=phs000001.v3.p1.
When available: With publication

Supporting Documents
Document types: Statistical/analytic code
How to access documents: We make the codes and models publicly available to the community via https://github.com/ncbi-nlp/deepseenet-plus.
When available: With publication

Additional Information
Who can access the data: researchers whose proposed use of the data has been approved
Types of analyses: The instructions are from the dbGAP repository
Mechanisms of data availability: The dbgap repository from NIH
